# Beyond pathogenesis: Detecting the full spectrum of ecological interactions in the virosphere

**DOI:** 10.1371/journal.pbio.3002109

**Published:** 2023-05-15

**Authors:** Stephanie D. Jurburg, Erik F. Y. Hom, Antonis Chatzinotas

**Affiliations:** 1 Department of Environmental Microbiology, Helmholtz Centre for Environmental Research—UFZ, Leipzig, Germany; 2 German Centre for Integrative Biodiversity Research (iDiv) Halle-Jena-Leipzig, Germany; 3 Institute of Biology, Leipzig University, Leipzig, Germany; 4 Department of Biology and Center for Biodiversity and Conservation Research, University of Mississippi, University, Mississippi, United States of America

## Abstract

The public perception of viruses has historically been negative. We are now at a stage where the development of tools to study viruses is at an all-time high, but society’s perception of viruses is at an all-time low. The literature regarding viral interactions has been skewed towards negative (i.e., pathogenic) symbioses, whereas viral mutualisms remain relatively underexplored. Viral interactions with their hosts are complex and some non-pathogenic viruses could have potential benefits to society. However, viral research is seldom designed to identify viral mutualists, a gap that merits considering new experimental designs. Determining whether antagonisms, mutualisms, and commensalisms are equally common ecological strategies requires more balanced research efforts that characterize the full spectrum of viral interactions.

## Abstract 2

Viral interactions with their hosts are complex and can be beneficial, yet the public perception of viruses is generally negative. In this Essay, the authors argue that new experimental designs are required to characterize the full spectrum of viral interactions, including mutualism and commensalism.

## A century of bad reputation

Historically, many microbes have been noticed primarily when they cause disease. This clear example of observational bias has skewed the perception of many viruses as “germs” to be avoided [1]. In 1898, Martinus Beijerinck first isolated the Tobacco Mosaic Virus and dubbed the substance *virus*, the Latin for “liquid poison,” jumpstarting both the field of virology and the negative reputation of viruses that persists today. Isolating most viruses for study has remained notoriously difficult, and to date, only a small fraction of all viral diversity (predominantly antagonists to the immediate host) has been studied in detail. However, sequencing-driven efforts, notably shotgun metagenomics, have allowed for the fruitful study of viruses with limited a priori knowledge or expectation of their genetic makeup or fitness impacts on potential hosts. An amazing diversity of viruses with distinct habitat preferences and distributions has been uncovered through sequence-based surveys [2,3], even within healthy human hosts [4]. Over 1% and up to 50% of non-rRNA transcripts in healthy invertebrates stem from RNA viruses and it is estimated that there are 10 to 100 times more viral particles than cells on the planet [5]. Despite sequence-based detection, much of the viral diversity of the world remains uncharacterized [5] and the ecological roles of this “viral dark matter” remain obscure [6,7]. For example, the ecological roles of single-stranded and double-stranded RNA viruses outside of disease scenarios are poorly understood [6]. Similarly, the functions of most viral proteins remain essentially unknown [8]. Moreover, although several mutualisms have been identified [9], highlighting the potential breadth of RNA viral interactions, many of these examples are not widely known.

**Fig 1:**
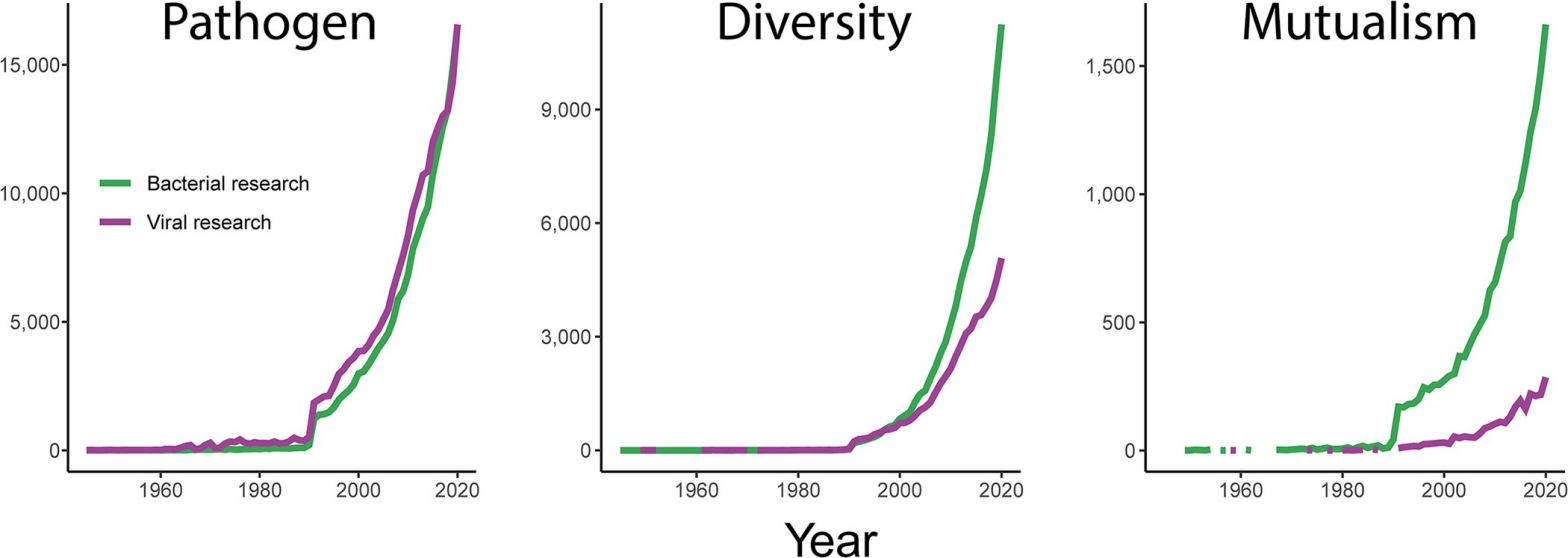
The pace of parasitism and mutualism-focused published research on viruses and bacteria over the last 3 decades. Mutualism, parasitism, and diversity-focused publications were identified through a keyword search for the terms mutualis* OR symbio*; parasite* OR pathog*; and divers*; respectively, AND bacteria* or viru* OR viro* OR vira*. The search was performed in Web of Science on March 29, 2023, and results were limited to “articles” from 2022 or earlier. From 1994 and 2000 onwards, more diversity-focused rather than parasitism-focused publications were recorded for both bacteria and viruses.

Newly discovered viruses likely have important ecological roles in the environments they inhabit [3,10]. Relative to an accelerating rate of viral discovery, few novel viral mutualists have been reported and existing reports of viral mutualists appear not to have altered the perception of viruses as solely pathogens within the broader life sciences [11]. As further viral diversity is uncovered, the lag in the identification of potential viral mutualists may become a growing gap in viral science (Fig 1). The legacy view of microbes as germs (with the exception of phage therapy [12]) has skewed the types of scientific questions asked, the research performed to answer them, and consequently, the translation of viral research into biotechnological applications. In the age of sequencing, bacterial research has increasingly focused on questions of diversity and beneficial or mutualistic interactions. Shedding their negative reputation has been harder for viruses than for bacteria, and research into viral diversity, and especially mutualisms, seems to have occurred at a slower rate (Fig 1).

Accelerating research into viral mutualists can shed light on viruses as bona fide ecological entities rather than strict antagonists and potentially broaden the biotechnological relevance of viral research. In this Essay, we discuss the challenge of identifying viral mutualisms and suggest experimental designs that can identify positive interactions. Balanced empirical designs may confirm or disprove whether viral antagonisms, mutualisms, and commensalisms are equally plausible ecological strategies in nature and help determine whether the general notion that viruses are predisposed to antagonism or are generally “bad” is justified. In turn, such research may accelerate the translation of viral research into biotechnological applications.

## Interactions in a context-dependent world

A key bottleneck in the identification of viral mutualisms is the inherent difficulty of defining these interactions [10]. Symbioses, or the shared genetic fate of two or more organisms via physical association [13], include a spectrum of interactions that may be dynamic and context specific, but broadly encompass different types of relationships: mutualism (in which all members exhibit fitness benefits), commensalism (one member benefits with no consequences for the other), and antagonism (one member benefits to the detriment of another). Viruses are obligate symbionts (i.e., they require a host to perform basic life functions) and while they were historically classified as parasites, the last decade of research has revealed the complexity of virus–host relationships. Often, virus–host relationships do not fit within traditional symbiosis categorizations [11]. Indeed, while clearly obligately dependent, the effect of most viruses (including phages) on the fitness of human hosts remains unknown [14].

Research to date shows that virus–host interactions can positively affect the evolution of the host species [15], expand the infected host’s phenotypic plasticity [16], and protect the infected host from pathogens [17,18]; however, such fitness benefits should be considered relative to defined time scales and with respect to abiotic and biotic contexts. Whether a host experiences negative, negligible, or positive fitness effects from interacting with a virus can depend on the temporal scale in question, in addition to the coevolutionary history of virus and host [19]. Viral antagonists have been shown to evolve into mutualists under strong selective pressures [20], revealing that ecological relationships can change, especially in the face of environmental change [13]. The scope of evolutionary history considered could also make a difference to the assessment of fitness impacts of a virus on a host. For example, the endogenization of retroviruses following infection has a negative effect on host fitness, but may promote adaptation over longer time scales by contributing viral genetic diversity to the host’s genome at the population level [15], and can directly alter the evolutionary trajectory of the host species. In more extreme cases, a virus may integrate with the host genome to form a new species [10]. Thus, it may be helpful to also consider the degree of partner genome integration [13] in descriptions and assessments of virus–host interactions.

Virus–host symbioses are asymmetric in partner dependence in a way that may profoundly shape viral transmission dynamics, coevolution, and stability along the mutualism–antagonism continuum [21]. As viruses are obligately dependent upon a host for reproduction, they must experience positive fitness effects in association with the host. Moreover, if host fitness is improved by a mutualistic virus, obligate dependence dictates that viral fitness (i.e., replicative, epidemiologic, or transmission fitness [22]) must also improve.

The abiotic context, or environment, can also influence the ecological outcome of viral partnerships [10,11], and conversely, viral partnerships can influence how the host experiences the abiotic context. Extended phenotypic plasticity, or tolerance to environmental fluctuations, has been documented for a wide range of virus-infected crops exposed to temperature extremes, flood, drought, and oxidative stress (e.g., [23] reviewed in [16]). The fitness outcome of these interactions has been generally shown to be context dependent. In one case, the cucumber mosaic virus was shown to have positive, neutral, or negative effects on the fitness of the model plant *Arabidopsis thaliana* depending on the host’s genotype as well as on light availability and temperature [24]. Because viral infection improves the plant’s tolerance to environmental stress, this virus is only beneficial under environmental extremes. Similarly, experimental drought conditions can result in the transition of the turnip mosaic virus from being pathogenic to mutualistic, promoting a higher rate of plant survival [20]. These fitness benefits can also act simultaneously on several ecological levels. While for the cases above the virus incurs direct fitness benefits on the host, in the case of the densovirus that infects the rosy apple aphid, the virus negatively affects the reproduction of the aphid host but induces a beneficial wing morph that improves dispersal and overall host survival [25]. Viral infection in this case has a negative effect on the fitness of individuals in the short term, but a potentially positive effect on aphid populations over greater time scales.

## Ecology’s nesting doll problem

**Fig 2:**
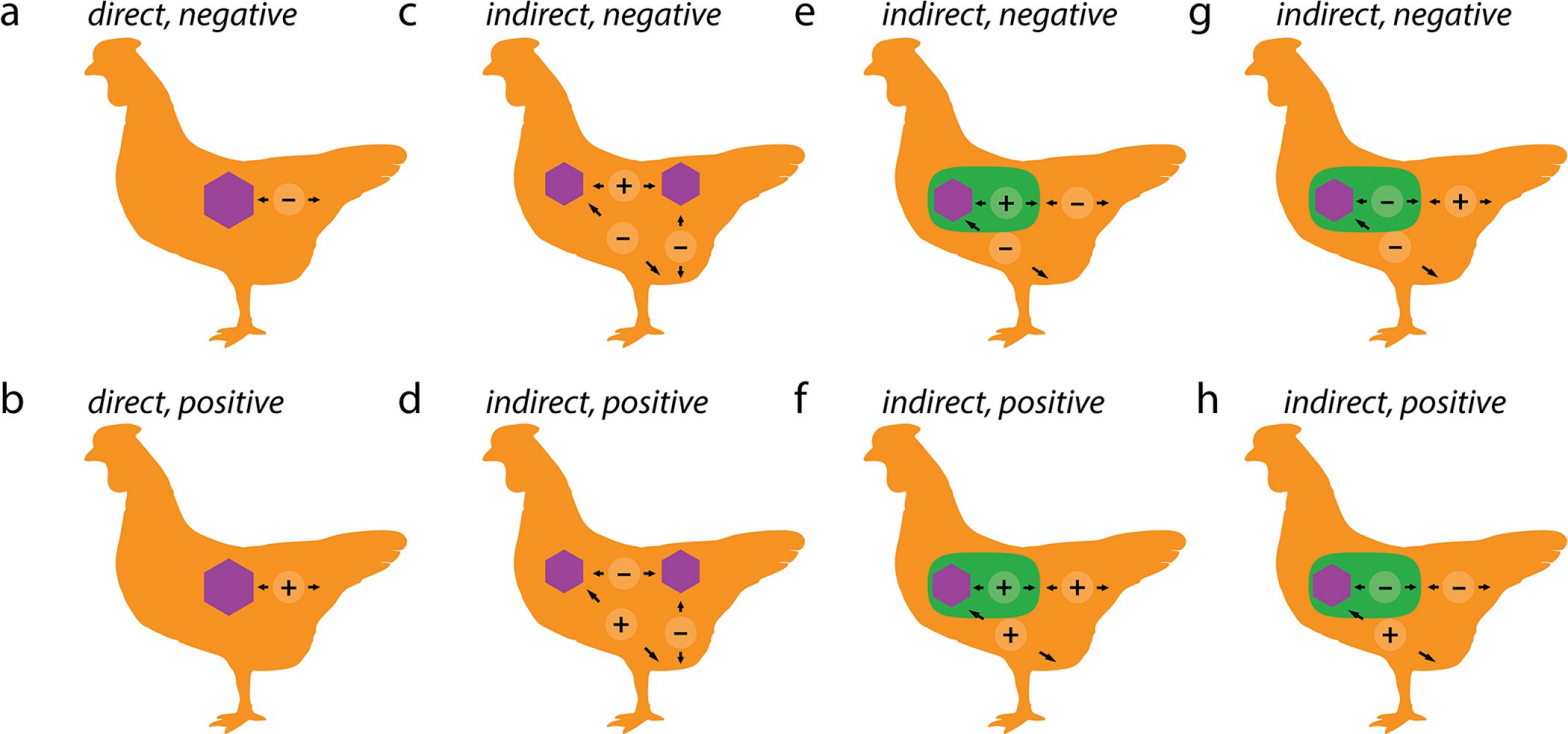
Studying viral interactions requires considering host nestedness. In their simplest form, direct interactions between a virus and a primary host (a purple hexagon and a chicken, respectively) can be on average negative (a) or positive (b) for the host. However, viral interactions often act on different levels of host nestedness, resulting in multiple simultaneous interactions (c–h). Within their primary host, viruses interact among each other, modulating their indirect relationship to the host (c, d). For example, infection by a nonpathogenic strain reduces the infection success by similar, pathogenic strains (e.g., superinfection exclusion, d). Viruses may also infect symbionts (a bacterial host, in green) that inhabit larger, secondary hosts, resulting in hierarchical nested interactions (e–h). Not shown explicitly but increasingly studied are viruses of fungi, viruses of microbial eukaryotes, viruses of viruses, and nested virus–virus interactions within a primary host within another, secondary host, and the indirect effects that viruses can have on the host’s environment.

Another layer of complexity is the potential nesting of viruses within multiple hosts (i.e., a phage within a bacterium in the animal gut). Ascribing a fitness measure to such hierarchical, interdependent interactions is complicated (Fig 2), particularly when based on molecular data, where interactions are seldom observed in situ and are instead inferred. In one case, viruses inhabiting parasitoid wasps improve the wasps’ ability to parasitize other insects [26]. In this example, the virus may be a direct mutualist of the wasp, but as a consequence, be an indirect antagonist of the secondary insect host.

Because multiple viruses can interact with each other within a host, and each may independently alter the relationship between the virus’s primary and secondary hosts, determining the nature and net outcome of a symbiosis may require considering multiple scales of host nestedness along with simultaneous, interdependent interactions (Fig 2) [27]. Coinfection (i.e., the infection of a host by multiple pathogens) of bacterial hosts by multiple phages may affect up to half of all bacterial cells [7,28], and novel, complex virus–virus interactions are being actively discovered [29,30]. For example, virophages are viruses of the *Lavidaviridae* family that infect giant viruses, which in turn infect microbial eukaryotes (protists) with varied fitness effects on the primary and secondary hosts [31]. The extent to which virophages influence the ecology of their hosts is currently unclear, although theoretical models suggest that this nested interaction influences the population dynamics of both the primary virus host and the secondary eukaryotic host [32]. So-called “zombie viruses,” are defective, virus-like, incomplete genomes that compete with full-genome relatives for reproductive success as virus “hitchhikers” and can reduce virus-associated host disease [33]. Harnessing zombie viruses as antivirals or adjuvants that functionally antagonize infections by full virus genome relatives to prevent disease (e.g., influenza [34], measles [35], and polio [36]) highlights the importance of understanding intra-viral interactions.

The outcome of infection can depend on a wide range of factors, including the mode of transmission and genetic relatedness among infecting viruses [37]. Given the multiple levels of hosts involved, viral mutualisms should be clearly defined relative to a specific host and virus, while considering other individuals that may mediate this relationship. In humans, colibactin, a genotoxic compound produced by enterobacteria in the gut that is associated with colorectal cancer, was recently shown to cause cell lysis by triggering prophage induction [38], situating viruses as intermediary actors in bacterial competition with negative consequences for the secondary host. In contrast, the *Curvularia* thermal tolerance virus is an integral part of a tripartite mutualistic symbiosis between the endophytic fungus *Curvularia protuberata* and the *Dichanthelium lanuginosum* grass, enabling all members to withstand temperatures of 65°C [39]. Similar dynamics have been theoretically predicted among the microbiota of freshwater metazoan *Hydra vulgaris*, in which infection of members of the resident bacterial community by a prophage promotes coexistence among the bacteria, which in turn can protect their host from fungal infections [40]. Understanding these complex interactions can have practical ramifications. For example, individuals coinfected with GB virus C and HIV exhibit slower progression to AIDS and higher survival rates than patients infected only with HIV [41]. Similarly, superinfection exclusion, in which an established viral infection protects the host from infection by other strains, has been used to protect crops from more virulent strains by preemptively infecting them with milder strains, but can also explain why viral vaccines often fail in infected individuals [42]. More generally, phage therapy leverages the antagonism between phage and target pathogenic bacteria to improve host fitness [12]. Similarly, zombie viruses can effectively neutralize SARS-CoV-2 viral infections, potentially offering a new therapeutic avenue for treating viral diseases and countering emerging pandemic threats [43]. In order to further such applications, virus–host relationships must be considered within broader contexts. As it has been broadly established that symbioses can shift from mutualisms to antagonisms and vice versa over time, and that they are influenced by environmental selection, mode of transmission, and the identity of hosts, probing virus–host relationships across a gradient of each of these factors may be necessary to determine their stability [44].

Direct positive interactions between viruses and eukaryotic hosts, and indirect/cascading interactions between viruses and host-beneficial bacteria (Fig 2A and 2D), remain underexplored both fundamentally and as a mode of biotechnological innovation, even though they are increasingly reported. Latent herpesviruses and astroviruses can protect animal hosts against bacterial infections by stimulating the host’s immune system [17,18]. By contrast, direct positive interactions among phages and bacteria can ultimately have negative effects on the encompassing host, as is the case with many “phage morons,” which are genes carried by phages that can confer additional fitness traits such as antibiotic resistance, virulence, or adhesion on their potentially pathogenic bacterial hosts [45]. For example, phage-encoded toxins are responsible for the high pathogenicity of *Vibrio cholerae*, *Escherichia coli*, and *Staphylococcus aureus* [10]. Identifying such nested interactions requires moving beyond classical pairwise interaction frameworks.

Importantly, viruses may also have a direct role in sustaining ecosystem services. Recent work in marine systems has reported that auxiliary metabolic genes acquired by viruses from hosts can perform vital functions at an ecosystem level. These include viral ammonia oxidizing genes from archaeal hosts (*amoC*) as well as genes involved in marine sulfur cycles that contribute directly to global nutrient cycling [3]. Phages, and to a greater extent archaeal and eukaryotic viruses, contribute to carbon cycling in marine systems through virus-mediated lysis [46,47], which can account for a substantial portion of carbon released (up to a third of the microbial biomass released yearly). One study discovered a lineage of giant viruses that may allow their unicellular eukaryotic hosts to obtain energy photosynthetically [48], while another found auxiliary metabolic genes responsible for the synthesis of bacterial phospholipids and nucleotides in marine viruses, which likely contribute to improving the fitness of bacterial hosts [49]. The authors also found evidence of viral genes that had been actively host-selected, suggesting the long-term persistence of these interactions. The link between ecosystem-level viral functions and the individual hosts adds yet another critical layer of complexity to consider.

## Detecting virus–host interactions and their outcomes

Designing experiments that can detect viral mutualisms will likely improve our understanding of viral ecology by providing a more complete picture of viral interactions. Given the complexity of viral interactions and the diversity of outcomes for nested hosts and the environment, it is important to consider that not all viral mutualists are necessarily “good” for biotechnological applications, and that viral candidates for biotechnology may not be selected for their mutualistic interactions. Viral mutualisms may be observed as positive fitness effects on both interacting partners as a result of this interaction. It is notoriously difficult to detect viral interactions from sequence data, as such data may only provide a snapshot of all the viruses in a sample at a given instance of sampling and does not directly link viral genes to their hosts. Available bioinformatic tools to predict virus–host interactions rely on shared molecular signals among virus and host (e.g., sequence matches between virus and host genomes or virus matches to host-encoded CRISPR spacers [50]) or a combination of these techniques [51]. However, the accuracy of these approaches depends on the representation of both phages and potential hosts in sequence databases, which is uneven and skewed towards pathogenic viruses. Continued sequencing and analysis efforts are essential for populating sequence databases with information about the lesser known viral groups (e.g., archaeal viruses [3] and virophages [28]). Bioinformatics methods can help identify virus–host relationships [50], but do not measure the effect of these interactions on host fitness. While charting the network of connectivity is an important first step, it is also imperative to elucidate the ecological effects or “directionality” and dynamism of these interactions. New combinations of technologies may further revolutionize our perspective on viral interactions. For example, combining phage-specific fluorescence in situ hybridization with microscopy and bioinformatics recently revealed that marine sponges control their associated microbiome by modulating phage populations [52].

**Fig 3:**
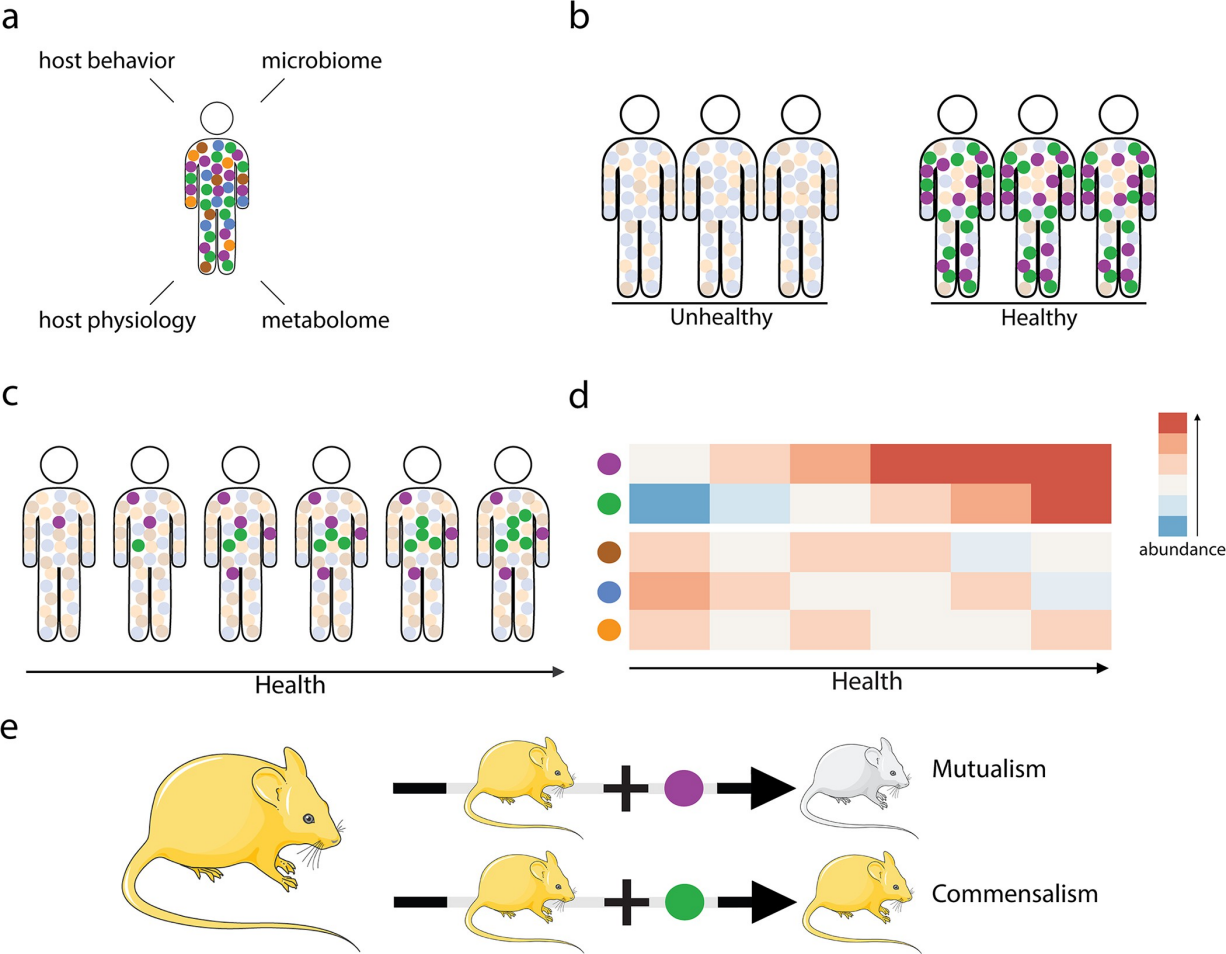
Detecting the full spectrum of virus–host interactions requires dedicated experimental designs. Health and fitness are multivariate and can be measured by various parameters (a). In large cohort studies, mutualistic viruses (purple and green circles) could be expected to be consistently present and abundant in healthy hosts (b, right) and could be absent or less abundant in unhealthy hosts (b, left). In longitudinal studies, the abundance of mutualistic viruses could be expected to increase gradually over time as the host recovers from disease (c) and to be positively correlated to host health (d). Distinguishing between mutualistic and commensal viruses will likely require isolating viral strains of interest and transplanting them into experimental systems (e) to directly assess whether they improve host health (purple circle, white mouse) or whether they have no net effect (green circle, yellow mouse). Mouse images were obtained from Smart Servier Medical Art (https://smart.servier.com/).

Identifying viral mutualists requires carefully crafted experimental designs that explicitly consider the biotic and abiotic contexts in which the interactions occur and that result in a defined fitness outcome (Fig 3). We believe measurements across a continuum of host fitness may be essential to identifying viral mutualists. Specific fitness metrics must be well chosen, as host fitness can be measured along several dimensions (e.g., behavior, metabolism, and physiology; Fig 3A). Importantly, the choice of fitness measurement may dictate whether or not viral mutualists can be detected, as their presence may affect only one aspect of host health but not another [53]. Some trial and error may be required in discerning and formulating useful and practical fitness metrics, and these will likely need to account for phenotypic outcomes integrated over the life histories of the (nested) hosts.

Conceptually, identifying viral mutualists within healthy hosts is expected to be more challenging than identifying pathogens based on a “contrast-to-noise ratio” argument: the decrease in fitness of a healthy individual due to a pathogen is often more easily detected than the increase in the fitness due to a mutualist in a healthy host (i.e., detecting pathology is easier than detecting the difference between “healthy” and “more healthy”). Here, available methodological approaches that have been previously applied to bacteria–host relationships [21] may be applied to virus–host relationships to yield novel insights. Machine learning algorithms may also serve to predict viral interactions, especially as our basic understanding of viral biology continues to expand and can serve to further inform these algorithms [8]

Explicitly seeking a broad range of “healthy” hosts can facilitate this by providing a gradient or spectrum of healthy states to examine. For large sample sizes, it is expected that viral mutualists will be consistently present (or persistent [9]) and abundant in healthy hosts and more abundant in healthier hosts (Fig 3B). While it may be impossible to distinguish between mutualists and commensals in this case, we see this sort of correlative analysis as a critical first step, especially as large viral survey datasets become more common. While linking viruses to hosts in sequence data is in its infancy [54], sequence-based studies have started to reveal the link between decreased viral richness and decreased host health. Lower viral diversity has been found in humans with type 1 diabetes [55] and metabolic syndrome [54], and studies employing mouse models have highlighted the role of viruses in promoting homeostasis in healthy hosts [56,57]. At the same time, other studies have begun to show that increased viral diversity may result in dysbiotic gut microbiota, which in turn can trigger inflammatory bowel disease in humans [58]. Using existing sequencing-based methods, incomplete or defective viral genome variants could be monitored that are often ignored or discounted; it may be fruitful to carefully inventory and analyze these viral lineages given our emerging understanding of zombie viruses [33].

Subsequently, longitudinal studies of recovering hosts that combine bioinformatics and ecological analyses may be useful in disentangling complex ecological relationships between viruses and hosts [59]. By monitoring the microbial and viral community in the gut of children, an 18-week longitudinal study found that phage diversity in infants was negatively related to the effectiveness of rotavirus vaccines [60]. In a 6-week longitudinal comparison of the rhizosphere soils of healthy tomato plants and plants infected with the pathogenic bacteria *Ralstonia solanacearum* in the field, the rhizosphere soils of healthy plants were found to have a higher abundance of *R*. *solanacearum*-specific phages, highlighting the role of phages in mediating the relationship between their primary and secondary hosts [61]. Through isolation of rhizosphere phages and bacteria, along with follow-up greenhouse experiments, the authors further confirmed that these phages endowed bacterial antagonists of *R*. *solanacearum* with a competitive advantage—a pattern they first identified through network analyses of the metagenomic data.

Indeed, discriminating between mutualists and commensals will likely require isolating viral strains or consortia and systematically testing their effects in experimental systems of varying complexity [62], including tissue cultures, reactors, and model organisms. By studying the effects of selectively adding bacteria or viruses to more simplified experimental systems outside of the original host, it may be possible to tease apart the relationship between bacteria and viruses in affecting host health. For example, in one notable case, combining the findings of experiments with germ-free mice and dendritic cells demonstrated that exposure to phage DNA alone was sufficient to trigger immune responses [63]. Simplified experimental systems reduce ecological complexity, facilitate higher sample sizes, and improve replication with greater control. They also offer opportunities to control trophic nestedness and explicitly test different environmental contexts in which target interactions are mutualistic [24].

## Conclusion

A deeper understanding of viral symbioses may serve as a first step towards identifying candidate viruses for biotechnological applications, but other systems-based approaches may be necessary to select candidate viral genes and proteins with high biotechnological potential [64]. In this realm, improved sequencing (i.e., coverage, read length, and assembly algorithms) has altered how we view the ecological role of viruses by facilitating the placement of genes within the context of a viral genome, particularly in the study of auxiliary metabolic genes in host fitness and their contribution to ecosystem services [65]. Even with our currently limited view, viruses have already demonstrated their tantalizing potential in mitigating human disease [66], promoting host adaptation to environmental change (reviewed in [20]), and promoting nutrient cycling [65]. Applied industries may benefit from identifying viruses that have broader positive effects on the host. For example, recent work has shown that viruses can, in general, expand the range of host stress tolerance [16,23].

The viral world remains a massive reservoir of untapped genetic diversity [65] and viruses are key ecological drivers in all environments [3,4]. A better understanding of virus–virus and virus–host interactions will be fundamental for developing more sophisticated virus-based technologies that aim to improve host fitness and next generation microbial management technologies to replace or complement antibiotics [12,67]. How to do so responsibly will require acknowledging the ecological complexities of viruses and rethinking experimental designs to capture the full spectrum of interaction types across different environments and hierarchies of biological organization at the individual, population, and ecosystem level.

While the obligate dependence of viruses on their hosts de facto dictates that they must benefit from the relationship, this dependency does not a priori dictate the costs or benefits on host fitness. The current scientific literature has been grossly skewed towards the viral antagonists, reflecting our historical and implicit prejudice about viruses causing disease. As the fields of viral ecology and environmental viromics (i.e., studying all the viral molecules in an environment) continue to grow, fueled by technological advances and accelerated by the COVID-19 pandemic, characterizing the full spectrum of ecological interactions of viruses must be a priority. Only then will it be possible to assess whether and to what degree obligate dependency predisposes viruses towards specific ecological strategies, whether the nature of viral interactions is dependent on host phylogeny, and whether the negative reputation of viruses is warranted.
